# Population structure and minimum core genome typing of *Legionella pneumophila*

**DOI:** 10.1038/srep21356

**Published:** 2016-02-18

**Authors:** Tian Qin, Wen Zhang, Wenbin Liu, Haijian Zhou, Hongyu Ren, Zhujun Shao, Ruiting Lan, Jianguo Xu

**Affiliations:** 1 State Key Laboratory for Infectious Disease Prevention and Control, National Institute for Communicable Disease Control and Prevention, Chinese Centre for Disease Control and Prevention. Beijing, 102206, China; 2 Collaborative Innovation Centre for Diagnosis and Treatment of Infectious Diseases, Hangzhou, 310003, China; 3 Novogene Bioinformatics Technology Co. Ltd, Beijing, 100083, China; 4 School of Biotechnology and Biomolecular Sciences, University of New South Wales, Sydney, 2052, Australia

## Abstract

*Legionella pneumophila* is an important human pathogen causing Legionnaires’ disease. In this study, whole genome sequencing (WGS) was used to study the characteristics and population structure of *L. pneumophila* strains. We sequenced and compared 53 isolates of *L. pneumophila* covering different serogroups and sequence-based typing (SBT) types (STs). We found that 1,896 single-copy orthologous genes were shared by all isolates and were defined as the minimum core genome (MCG) of *L. pneumophila*. A total of 323,224 single-nucleotide polymorphisms (SNPs) were identified among the 53 strains. After excluding 314,059 SNPs which were likely to be results of recombination, the remaining 9,165 SNPs were referred to as MCG SNPs. Population Structure analysis based on MCG divided the 53 *L. pneumophila* into nine MCG groups. The within-group distances were much smaller than the between-group distances, indicating considerable divergence between MCG groups. MCG groups were also supplied by phylogenetic analysis and may be considered as robust taxonomic units within *L. pneumophila*. Among the nine MCG groups, eight showed high intracellular growth ability while one showed low intracellular growth ability. Furthermore, MCG typing also showed high resolution in subtyping ST1 strains. The results obtained in this study provided significant insights into the evolution, population structure and pathogenicity of *L. pneumophila*.

*Legionella pneumophila* is an environmental organism and an important human pathogen causing nosocomial and community-acquired pneumonia^1^,^2^. *L. pneumophila* was first found to be associated with an infectious outbreak in 1976 in the United States^3^ and was later reported worldwide^2^. *L. pneumophila* infects humans to cause Legionnaires’ disease (LD) as well as a milder form known as Pontiac fever. LD is a potentially fatal form of atypical pneumonia with a case fatality rate ranging from 5% to 30%^4^. Approximately 90% of LD is caused by *L. pneumophila* serogroup 1^5^.

*L. pneumophila* is found in natural and man-made aquatic environments, such as potable water systems, spa water, and cooling towers^1^,^5^,^6^. Biofilm and Amoeba in the water systems provide optimal growth environment for *L. pneumophila* and is important for persistence and replication of *L. pneumophila*^7^,^8^. Transmission of bacteria from the environment to humans occurs via inhalation or aspiration of *L. pneumophila* -containing aerosols^9^,^10^.

As the vast majority of LD cases are caused by *L. pneumophila*, and this species is very common in the environment, strain differentiation using appropriate subtyping methods is necessary to identify the sources of contamination and determination of routes of transmission, together with population structure analysis to determine the genetic and epidemiological characteristics and pathogenic potential of *L. pneumophila*. A large number of subtyping techniques have been used for epidemiological typing purposes, including sequence-based typing (SBT) and pulsed field gel electrophoresis (PFGE), which typically take several days to obtain results^11^. SBT, which is commonly called multilocus sequence typing in other species, was developed by members of the European Working Group for Legionella Infections (EWGLI) and is a powerful method based on the sequencing of seven gene loci^12^,^13^. It is now considered the gold standard tool for *L. pneumophila* typing and a large SBT database is available. PFGE is a highly discriminative epidemiological subtyping tool for *L. pneumophila*^14^,^15^ and is the most commonly used method to investigate LD outbreaks and trace the environmental source of infection while SBT is the mostly used population structure analysis tool of *L. pneumophila.* However, the discriminatory power of SBT could not meet the need for distinguishing outbreak isolates or non-outbreak isolates^16^,^17^,^18^. Recently, whole genome sequencing (WGS) has been applied to subtype various pathogens including *L. pneumophila*. For *L. pneumophila* subtyping, WGS-based methods provided better discriminatory power to distinguish outbreak isolates from non-outbreak isolates^17^,^19^,^20^,^21^.

The use of whole genome sequencing for epidemiological typing is generally based on either whole genome SNP comparison or gene by gene comparison^21^,^22^. We recently proposed the concept of minimal core genome (MCG) typing^23^. MCG is defined as the non-repetitive genes present in all strains of a species. Using *Streptococcus suis* as a model pathogen, the application of MCG typing in combination with population structure analysis divided *S. suis* into 8 MCG groups, one of which is highly pathogenic^23^. The MCG SNPs allow rapid differentiation of different MCG groups of *S. suis*^24^. In this study we applied MCG to *L. pneumophila*.

In a previous study, we used SBT to assess the subtyping characteristics and population structure of *L. pneumophila* strains isolated from water systems in China and revealed that there were several clonal groups, some of which were also prevalent in other countries^25^. In this study we sequenced a subset of this set of well characterized isolates to develop a minimum core genome (MCG) typing scheme for subtyping and population structure analysis of *L. pneumophila*. We show that MCG typing divides *L. pneumophila* into nine MCG groups, one of which was found to have lower intracellular growth ability.

## Results

### General features and gene content of *L. pneumophila* genomes

Using Illumina high-throughput sequencing, we sequenced 44 *L. pneumophila* isolates which represented 37 sequence types (STs) and 12 serogroups. Nine completed genomes obtained from the NCBI database were also included in the analysis. The genomes of 53 *L. pneumophila* strains were relatively conserved in genome size, G+C content, number of protein-coding ORFs and gene size (Table S1). The number of genes per strain ranged from 2,926 to 3,490. All genomes have approximately the same codon usage frequency.

To assess the gene content among the genomes, putative genes from all the genomes were grouped into clusters where each cluster member is homologous to one another. The clusters represented proteins shared between the genomes, and the presence of a member within these clusters for a particular strain represents the existence of the gene for this protein within the genome of that strain. There were a total of 6,837 clusters (pan genome) of 53 genomes, and 2,093 clusters (core genome) that contained members from every strain sequenced. The number of strain-specific genes varied from 0 to 199 genes, with strain SH003 having the largest number of strain-specific genes. There was no strain-specific gene observed in three strains, Hu6, WD_4_1102a, and ATCC43290.

### Maximum likelihood analysis using single-copy orthologous genes (core genes)

**Figure 1:**
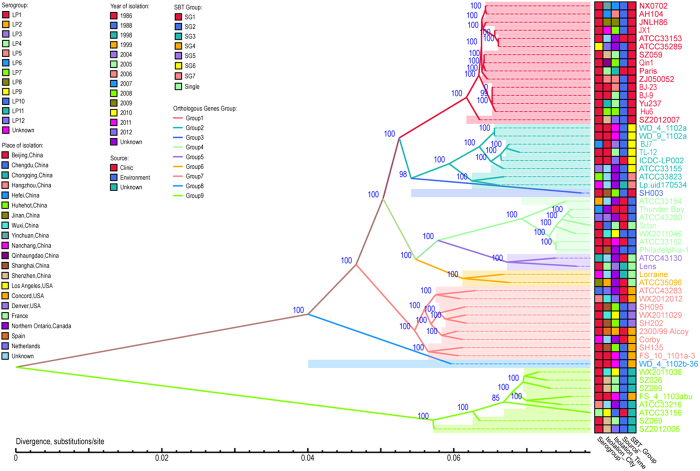
Maximum likelihood tree based on 1896 single-copy orthologous genes identified in 53 *L. pneumophila* genomes. The strains appear to belong to nine different serogroups (marked in different colors). The strain information, including serotype, isolation city, isolation time and source, are shown in the box with different colors. This phylogenetic tree was rooted using *Legionella dumoffii* strain Tex-KL as an outgroup, although the outgroup was removed from the tree. See Figure S1 for the rooted tree with scaled branch lengths.

Among the 2,093 core genes, 1,896 genes were single-copy orthologous genes. A maximum likelihood tree of 53 *L. pneumophila* strains was constructed based on the 1896 single-copy orthologous genes (Fig. 1). Nine groups (group 1 to group 9) were observed. Among them, two groups contained only one strain and the other seven groups each contained 2 to 15 closely related strains. In these groups, the strains were isolated from extended time periods and diverse locations, and belonged to different serogroups and sampling sources.

We attempted to identify genes specific to each group, but none was found. However we found marker genes that were different between at least two groups by allelic variation, although not specific to a single group. The marker genes are present in all strains and vary in sequence and the numbers of alleles of the 25 markers genes are between 2 and 9 (Table S2). There are 25 marker genes identified of which 19 have known functions while the other six encode hypothetical proteins (Table S2).

### Identification of the minimum core genome SNPs and MCG groups

By comparison to the reference genome of strain Philadelphia 1, we found 323,224 SNPs among the 53 isolates, of which 314,059 SNPs were in the recombination regions and were excluded. The remaining 9,165 SNPs were referred to as MCG SNPs. The pairwise number of SNP differences between strains was between 0 and 3,365, and the coverage number of SNP difference was 1,611 SNPs.

We used the Bayesian statistics tool STRUCTURE^26^ to reveal the population structure of *L. pneumophila* and to establish population genetics-based subdivisions of the species for strain identification and typing using MCG. By testing subdivisions of the 53 *L. pneumophila* isolate into between two and 15 subpopulations, we found that the optimal number of subpopulations was nine, with 15, 7, 1, 7, 1, 1, 9, 1 and 8 isolates being assigned to subpopulations 1 to 9, respectively (Table S3). However, three strains (ATCC43130, ATCC35096, Lp.uid170534) were not assigned to a group. Based on phylogenetic analysis we manually assigned them to a group as described below.

**Figure 2:**
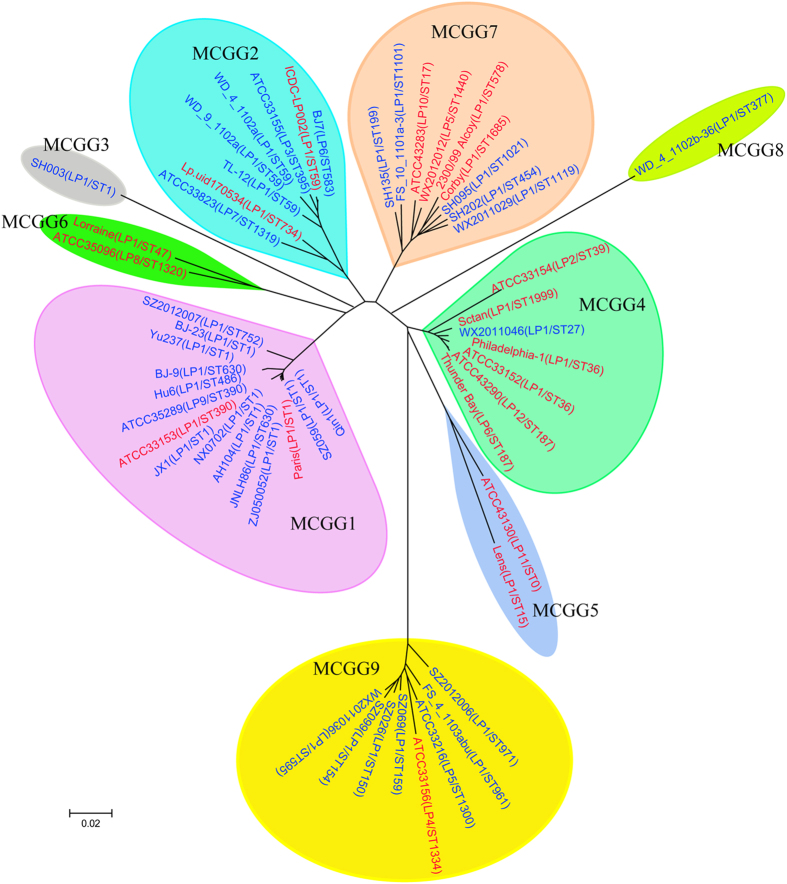
Phylogenetic tree and MCG groups of 53 *L. pneumophila* strains based on 9165 MCG SNPs. The serogroups and/or SBT types (STs) are represented in brackets. Strains from patient and environmental water samples are shown in red and blue, respectively.

Phylogenetic analysis was also performed using both the neighbor-joining algorithm and the minimum evolution algorithm^27^,^28^. The clustering of the isolates was largely consistent with the STRUCTURE analysis (Fig. 2). MCGG1, MCGG3, MCGG4, MCGG7, MCGG8 and MCGG9 exactly matched Subpopulations 1, 3, 4, 7, 8 and 9, respectively. In the STRUCTURE analysis, strain Lp.uid170534 was assigned as ungrouped; however, in the neighbor-joining tree, Lp.uid170534 was clustered together with strain ATCC33823 (Subpopulation 2). Thus we assigned Lp.uid170534 to MCGG2 because of these two strains were clustered together in the phylogenetic tree. Additionally, ATCC43130 was clustered together with Lens, forming MCGG5; ATCC35096 was clustered together with Lorraine, forming MCGG6. The grouping of the isolates based on 9,165 MCG SNPs were consistent with the clustering based on 1896 single-copy orthologous genes (Figure S2).

We computed the genetic distances for all of the combinations of within- and between-group comparisons where the within-group distance is 2.1 to 13.3 times smaller than the between-group distances. The smallest within-group distance was 0.012 for MCGG 1, which is 13.3 times less than the distances between MCGG1 and the other MCGGs. The within-group distances ranged from 0.012 to 0.095, which are smaller than the between-group distances (range of the 0.106 to 0.317). Especially the within-group distance of MCGG1, MCGG2, MCGG4, MCGG7 and MCGG9 is much smaller than the between-group distance (Table S4), indicating considerable divergence between MCG groups.

MCGG1 contained 15 strains, seven of which are ST1 and two and four are single-locus variants (SLVs) and double-locus variants (DLVs) of ST1. However one ST1 strain SH003 was assigned to MCGG3; both gene content and single-copy orthologous gene trees placed SH003 away from the other ST1 strains. MCGG2 was constituted by five serogroup 1 strains with four being ST59 and one being ST734 (shared three loci with ST59), and one serogroup 3 strain of ST395 (shared five loci with ST59), one serogroup 6 strain of ST583 (shared four loci with ST59) and one serogroup 7 strain of ST1319 (shared four loci with ST734). MCGG4 contained four serogroup 1 strains (two ST36 strains, one ST27 strain and one ST1999 strain) and one each of serogroup 2 (ST39), serogroup 6 (ST187) and serogroup 12 (ST187) strains. ST27, ST36 and ST39 are DLV, triple-locus variant (TLV) and TLV of ST187, respectively. ST1999 shares two loci with ST187. MCGG5 contained 2 strains, Lens (serogroup 1, ST15) and ATCC43130 (serogroup 11). They shared one locus. MCGG6 contained ATCC35096 (serogroup 8, ST1320) and Lorraine (serogroup 1, ST47). They shared two loci. MCGG7 contained seven serogroup 1 strains from seven STs, one serogroup 5 and one serogroup 10 strains. Among the seven serogroup 1 strains, four belonged to SBT group 4 and three belonged to SBT group 5. The serogroups 5 and 10 strains belonged to SBT group 4. MCGG8 contained a single serogroup 1 strain, WD_4_1102b-36 (ST377, SBT group 4). MCGG9 contained six serogroup 1 strains, one serogroup 4 (ATCC33156) and one serogroup 5 (ATCC33216) strains. Five of the six serogroup 1 strains in MCGG9 belonged to SBT group 3, and another belonged to SBT group 4. ATCC33156 and ATCC33216 belonged to SBT group 3.

There is a good concordance between MCG groups and SBT groups. MCGG1, MCGG 4 and MCGG 9 correspond to SBT group 1, SBT group 2, and SBT group 3 respectively. MCGG 2 is inclusive of SBT groups 6 and 7. MCGG7 is inclusive of SBT groups 4 and 5. However, two exceptions were observed: SH003 and FS_4_1103abu. SH003 was a ST1 strain and was assigned as the sole member of MCGG3 as discussed above. FS_4_1103abu, an SBT group 4 strain, was assigned to MCGG8 and was not grouped with other SBT group 4 strains in MCGG7.

### MCG typing of ST1 strains

**Figure 3:**
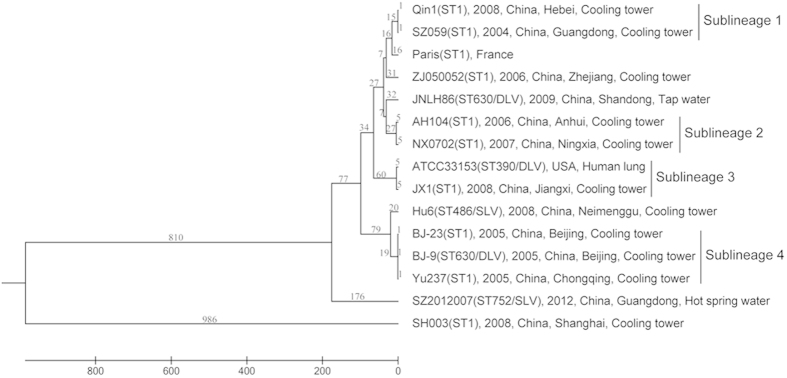
Phylogenetic tree of 10 ST1 serogroup 1 strains, 2 strains of SLVs (belong to ST486 and ST752) and 3 strains of DLVs (belong to ST390 and ST630) based on 9,165 MCG SNPs. The isolation year, country, province if from China, and source were listed after the strain names. The numbers on the branches were the number of SNPs on a given node.

ST1 is the most prevalent ST among both clinical and environmental isolates worldwide. ST1 strains and its SLVs and DLVs were separately evaluated for the discriminatory power of MCG typing. As seen in Fig. 3, the strain SH003 was the most divergent and was clustered far away from the other strains and was excluded from the distance analysis. The pairwise distances between strains were between 1 and 414 MCG SNPs, and the overall mean distance was 164 SNPs. There was minimal MCG difference between some strains from different countries. The French strain Paris and the US strain ATCC33153 clustered together with two (Qin1 and ZS059) and one (JX1) Chinese strains respectively. There were four sublineages (sublineages 1 to 4) observed with fewer than 10 MCG SNP differences. Each sublineage contained strains isolated from different countries or multiple provinces in China. However it should be noted that the number of genome SNPs is much bigger than the number of MCG SNPs as the latter subset of the former.

### Pan-genome tree analysis

**Figure 4:**
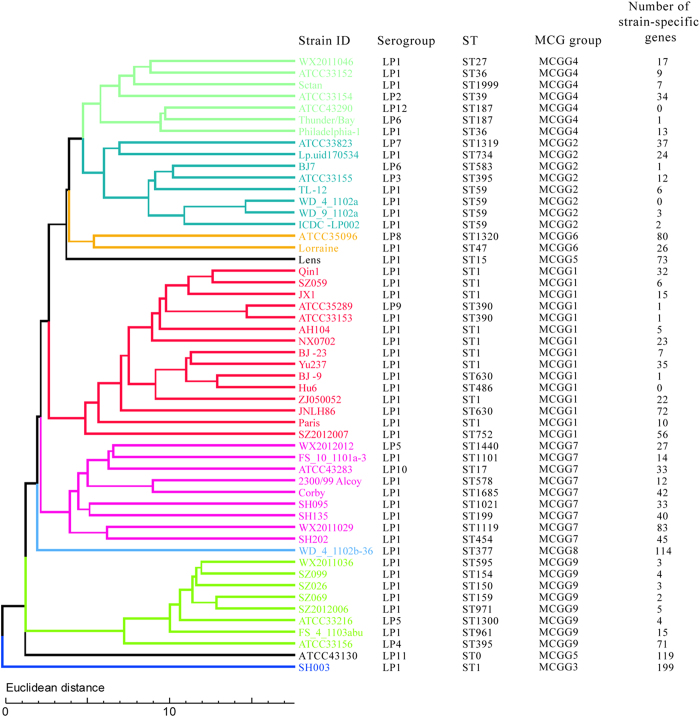
The pan-genome tree of 53 *L. pneumophila* strains based on the gene content (presence/absence of genes). Different MCG groups are marked by different colours. Strain name, Serogroup, SBT type (ST), MCG group, and number of strain specific genes are shown in the columns on the right.

A phylogenetic tree was constructed by using a distance matrix based on the presence or absence of genes among the strains (Fig. 4). The grouping of the 53 strains based on the gene content showed good concordance with MCG groups except that the pan-genome tree failed to cluster the two MCGG5 strains into one group. However, the clustering and relationships of strains within groups were different, suggesting that the pangenome tree has lost much of its phylogenetic signal due to lateral gene transfer events.

### Assessing difference in intracellular growth ability among the MCG groups

**Figure 5:**
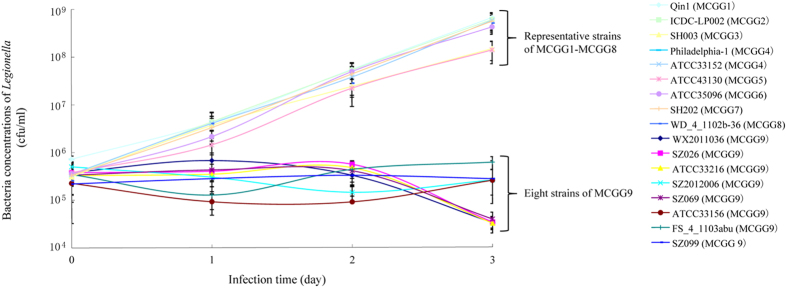
Intracellular growth ability of *L. pneumophila* strains of MCGG 9 and representative strains of other eight groups. The data of one strain from each group of MCGG1-MCGG8 and all eight strains of MCGG9 was shown.

With the robust division into MCG groups, we examined any difference between the groups in pathogenicity using intracellular growth ability as a marker which is a widely accepted index to assess the pathogenicity of *L. pneumophila*^29^. We compared the intracellular growth ability in J774 cells of the 44 *L. pneumophila* strains we sequenced and the reference strain Philadelphia 1. MCGG1 - MCGG8 strains had bacterial concentrations of 10^5^–10^6^ CFU/ml, 10^6^ CFU/ml, 10^7^ CFU/ml and 10^8^ CFU/ml on day 0, 1, 2 and 3 of infection, respectively, and showed no difference in bacterial concentrations to the strain Philadelphia-1 by the t test (p > 0.05). However, the MCGG9 strains had 10^5^–10^6^ CFU/ml or lower within three days’ infection and were significantly lower than that of Philadelphia-1 (p < 0.05) (Fig. 5).

We then investigated the genetic basis of this difference by comparing the genomes of MCGG9 strains with the other groups. There were no MCGG9 group specific genes or genes specific to all other groups. However there were 22 lethal mutations (premature stop, damaged start codon, damaged stop codon) associated with 19 genes in the genomes of the eight MCGG9 strains. The majority of the inactivated genes (15) encode hypothetical proteins while four are associated with protein transport and/or binding (Table S5). One gene, *katA*, has previously been showed to be with pathogenicity^30^. *katA* encodes a catalase-peroxidase and may affect the sensitivity to exogenous hydrogen peroxide. All MCGG9 strains were negative for catalase-production (data not shown).

## Discussion

In this study, we sequenced 53 isolates of *L. pneumophila* covering different serogroups and STs and developed a MCG typing method to divide the *L. pneumophila* population into subpopulations/groups. Several previous studies have used WGS to study the genomic diversity of *L. pneumophila* and to determine the power of WGS for outbreak investigation^21^,^22^. Underwood *et al.* used whole genome SNPs to compare with SBT for typing *L. pneumophila* and found that SBT is a good proxy to WGS to determine strain relationships^21^. Moran-Gilad J *et al* used outbreak isolates to define core genome MLST extending the MLST typing concept and found cgMLST has high resolution to differentiate outbreak clusters caused by *L. pneumophila*^22^. Our study further validated the power of WGS based typing for subtyping and population structure analysis of *L. pneumophila*. Our study differs from the previous studies by defining the minimum core genome and demonstrated the usefulness of MCG typing by differentiating *L. pneumophila* into nine MCG groups with potential difference in virulence and pathogenicity and other phenotypic characteristics. Our genome data extends the genomic diversity of *L. pneumophila.* Based on genome comparison, 1,896 genes were conserved in all strains which are fewer than those reported previously^17^,^20^,^21^, suggesting that our samples represented a wider breadth of the population than the samples in previous studies as the more diverse strains were sampled the more smaller the core genome becomes until it stabilizes^23^.

MCG typing was a recently proposed new bacterial genome typing method and was first developed using a zoonotic pathogen *S. suis*^23^. MCG refers to non-mobile genes that are shared by all strains of a given species^31^,^32^. Mobile genes were excluded from MCG, since these genes carry mixed phylogenetic signals. Additionally, SNP sites with a high frequency of recombination were removed from the core genome genes to increase the precision of the assignment of an isolate to a subpopulation.

Phylogenetic signals for inference of strain relationships may be obscured by recombination. Based on the core genome, we found that in the set of *L. pneumophila* isolates studied there were only 9,165 mutational MCG SNPs out of a total of 323,224 SNPs after filtering out recombinational SNPs. The ratio of MCG SNPs to total SNPs is significantly lower than that in the *S. suis* genome (58,501 MCG SNPs out of a total of 190,894 SNPs in *S. suis* core genome)^23^, suggesting that recombination is very frequent among *L. pneumophila* genomes, which is consistent with previous findings that recombination is a significant driver of the evolution of the *L. pneumophila* genome^33^,^34^.

Species is currently the lowest rank in bacterial taxonomy. However, in clinical care of patients, it is far more relevant to classify bacteria to a level that informs the mode of pathogenesis and the potential of the strain to cause severe disease, so that appropriate clinical care can be rendered. We showed in *S. suis*, the MCG groups may be used as taxonomical units to identify one of the MCG groups with potential to cause severe clinical infections and large scale outbreaks^23^. In this study, we applied the same approach to analyse the MCG groupings in *L. pneumophila* and can divide *L. pneumophila* isolates used into 9 MCG groups. Since these divisions are robust by both population and phylogentic analyses and difference in pathogenicity was observed between MCG groups, these MCG groups could also be considered as taxonomic units within *L. pneumophila*. These MCG groups can be easily differentiated using group marker genes (Table S2). The division is also biologically significant as we have found that MCGG9 is less capable of intracellular growth. Further studies of the difference among the MCG groups will enhance our understanding of the evolution of virulence of *L. pneumophila*.

ST1 is the most prevalent ST among both clinical and environmental *L. pneumophila* isolates worldwide^25^,^35^,^36^,^37^. In this study we included 10 ST1 isolates. All but one was grouped together. Additionally two SLVs (ST752 and ST486) and two DLVs (ST630 and ST390) were also grouped among the ST1 isolates. In particular two ST630 isolates were separated into different branches among ST1 isolates, suggesting independent origin of the two ST630 isolates. Considering the high level of recombination in *L. pneumophila,* it is not surprising that an ST arose through recombination independently more than once. The anomalous ST1 strain SH003 which is very distantly related to other ST1 strains is also likely to have arisen independently. The use of MCG SNPs which have been removed of recombinant SNPs provided a robust phylogenetic relationship of the isolates. We showed that isolates from different provinces of China and closely related isolates from other countries were found in several sublineages, suggesting wide spread distribution of such lineages. Further epidemiological studies of these lineages will be significant as it is possible that these wide spread clones are more likely to cause disease or outbreaks. Subtyping of ST1 strains using the MCG SNPs would be of significant value to understand the local and global spread of ST1 and its sublineages.

Invasion and intracellular replication of *L. pneumophila* within protozoa play major roles in the transmission of Legionnaires’ disease, and important relationships between the intracellular growth ability of *L. pneumophila* within protozoa and macrophages and human LD have been seen^33^,^34^. We found MCGG9 has lower intracellular growth ability suggesting that this group is less invasive and less likely to cause disease in humans. As shown in Fig. 1 and Figure S1, MCGG9 was the earliest diverged group, suggesting that other groups may have also gained pathogenicity during its evolution. However no unique genes were found in the latter groups. There could be other small genetic changes leading to increased pathogenicity. It is also possible that MCGG9 has partially lost its pathogenicity since several lethal mutations including one in *katA* in the genome of MCGG9 strains were observed. The inactivation of *katA,* encoding catalase-peroxidase, may affect the sensitivity to exogenous hydrogen peroxide and have been shown to affect the virulence of *L. pneumophila* in the THP-1 macrophage cell line^28^. However, MCGG9 may still cause disease as one of the strains was isolated from human infection.

In conclusion, our study defined the minimum core genome of of *L. pneumophila* from sequencing and analysis of 54 representative isolates and divided *L. pneumophila* into nine MCG groups. These groups can be easily differentiated using the MCG SNPs or MCG marker genes. We further found that MCG groups differ in ability to grow intracellarly, suggesting difference in virulence between MCG groups with MCGG9 having lower intracellular growth ability. We also showed that MCG SNPs can further divide ST1 isolates into multiple lineages with several lineages containing strains from different countries or different provinces within China. Better surveillance of these widely distributed sublineages will be significant in control and prevention of outbreaks caused by them. This study provided significant insights into the evolution, population structure and pathogenicity of *L. pneumophila.*

## Methods

### Sequenced strains and analyzed whole genome sequences

A total of 53 whole genomes of *L. pneumophila* were analyzed in this study. Forty-four isolates were newly sequenced for this study and the other 9 whole genomes (those of *L. pneumophila* strains Philadelphia-1, Thunder Bay, Paris, Lorraine, Lens, Corby, ATCC43290, 2300/99 Alcoy and Lp.uid170534) were obtained from the NCBI database. The ATCC33152 we sequenced, which is the same strain as Philadelphia 1, has been kept in our laboratory more than seven years and subcultured for more than 10 passages. All strains were epidemiologically unlinked. Of the 53 strains, 40 were serogroup 1 and 13 strains were other serogroups. The 53 *L. pneumophila* serogroup 1 strains as comprised of 37 STs of seven SBT groups and five singletons (Figure S3). The *neuA* locus in ATCC43130 was not detected, so the ST of this strain could not be determined and assigned as ST0 here. Metadata for the strains whose genomes were compared is presented in Table S6, including species, serogroups, dates and countries of isolation and GenBank accession numbers.

### DNA sequencing and assembly

Bacterial strains were sequenced using Illumina sequencing by constructing two paired-end (PE) libraries with average insertion lengths of 500 bp and 2,000 bp. Sequences were generated with an Illumina GA IIx (Illumina Inc., San Diego, CA, USA). Raw data was processed in four steps, including removing reads with 5 bp of ambiguous bases, removing reads with 20 bp of low quality (≤Q20) bases, removing adapter contamination, and removing duplicated reads. Finally, 100× libraries were obtained with clean paired-end read data. Assembly was performed using SOAPdenovo v1.05^38^.

### Genes predicted and Function annotation

Genes were predicted using Glimmer v3.02^39^. This software predicts start sites and coding region more effectively and has better interpolation of hidden Markov models, reducing the ratio of false positive predictions. Functional annotation was accomplished by analysis of protein sequences. Genes were aligned with databases to obtain the annotation corresponding to their homologs, with the highest quality alignment result chosen as the gene annotation. Functional annotation was completed by comparing BLAST v2.2.23 (http://blast.ncbi.nlm.nih.gov/Blast.cgi) results in the M8 format to the Kyoto Encyclopedia of Genes and Genomes (KEGG) v59^40^, Cluster of Orthologous Groups of proteins (COG) v20090331^41^,^42^, SwissProt v2011_10_19^43^, NR v2012-02-29, and Gene Ontology (GO) v1.419^44^ databases.

### SNP identification

We examined single-nucleotide polymorphisms (SNPs) through pairwise comparisons of *L. pneumophila* genomes using SOAPsnp^45^ and MUMmer^46^. For isolates with complete genome sequences, SNP selection was performed using the NUCmer program in the MUMmer package^46^. For SNP detection in the draft genomes, reads with low quality (>3 consecutive bases with a quality score of ≤Q20) were removed before SNP calling. SNPs were called if they met the following criteria using SOAPsnp^45^: (i) each SNP site was covered with ≥20 reads, (ii) ≥5 bp was the distance between two SNP sites, (iii) the SNP was not located in a repeat region, and (iv) the prior probability of heterozygous SNPs is ≤0.1%.

### Analysis of recombination and removal of recombinant SNPs.

Gene segments with recombination in the 53 isolates were identified using the method described by Feng *et al.*^47^. In the method we used, if the segments between two adjacent SNPs are defined as ISSs (inter-SNP segments), the distribution of ISSs around the genome is expected to follow an exponential distribution if all the observed SNPs are due to mutations that occur as a Poisson process. However, ISSs brought in by recombination events will disturb this distribution and form anomalous clusters of ISSs, which have shorter distances between SNPs due to imported segments carrying more SNPs. Therefore, the overall distribution observed with these ISSs will have an excess of short ISSs due to recombination and will not follow an exponential distribution. This excess of short ISSs may be removed to fit an exponential distribution if most parts of the genome have not been involved in recombination. A progressive exclusion of the short ISSs will allow one to find a cutoff value to fit an exponential distribution and to identify and remove ISSs due to recombination.

Since there was a large amount of recombination in *L. pneumophila*^31^, we did not remove the whole gene where recombination was detected; instead, only relevant portions of the recombined regions were removed. As such, the phylogenetic content is more likely to reflect the evolutionary history of vertical descent in populations and their true relationships.

### Population structure analyses

The program STRUCTURE 2.2^48^ was used to analyze the SNPs in the 53 isolates at the genome level, assuming one to 15 populations for five iterations each, using the admixture model and uncorrelated allele frequencies. A burn-in of 50,000 replications was discarded, and 150,000 additional replications were analyzed. The burn-in period was sufficient to stabilize log-likelihood values. Each value of K is based on the run with the highest likelihood value. Likewise, a standard measure of genetic distance, Fst, was calculated from the STRUCTURE 2.2 run with the highest likelihood value for K at 7 (Table S7) and showed the divergence of each population from the estimated ancestral allele frequencies. An admixture model and independent allele frequency were used for STRUCTURE analysis. The assignment of an isolate to a subpopulation was based on the largest percentage of ancestry contained in an isolate.

### Phylogenetic analysis

Core-pan gene clusters were constructed using genes from all 53 *L. pneumophila* strains. The current analysis focused on single copy gene families, which are determined by aligning protein sequences via BLAST. Gene presence in the same gene cluster was defined as a match with 70% sequence identity and 50% of sequence coverage^49^. In addition, the copy number of a gene in each strain was calculated. The clusters were used to generate a matrix of 1 and 0s, corresponding to the presence or absence of a gene in each of the strains. This matrix was used as the input for a parsimony analysis, which generated a tree with the most parsimonious representation of the data.

A phylogenetic tree based on concatenated sequences of single-copy orthologous genes was constructed using the maximum likelihood method with Treebest software^50^. A phylogenetic tree based on the core genome SNPs was constructed using the neighbor-joining or minimum evolution algorithms in MEGA^51^. Bootstraps were performed with 1,000 replicates. The program MEGA was also used to calculate the *p*-distance (*p* = *n*_*d*_/*n*) within and between population groups, where *n*_*d*_ is the number of sites with difference and *n* is the total number of sites.

### Intracellular growth assay

The *L. pneumophila* strains were revived from lyophilized stocks. The bacteria were streaked onto buffered charcoal yeast extract (BCYE) agar plates, and one typical colony of each strain was picked up and inoculated onto another BCYE agar plate. One loopful of bacterial lawn was picked up and inoculated onto buffered yeast extract (BYE) broth at 37 °C until they reached early stationary phase. Approximately 2 × 10^9^ bacteria were pelleted, resuspended and diluted (1:1,000) in Roswell Park Memorial Institute (RPMI) 1640 tissue culture medium. The bacteria were then added to J774 cells or guinea pig peritoneal macrophages (2 × 10^5^ per well) in 24-well dishes to give a multiplicity of infection (MOI) of approximately 10. The infected cells were incubated at 37 °C under 5% CO_2_-air for 1.5 h and then washed three times with PBS to remove extracellular bacteria. To measure bacterial internalization, 1 ml of sterile distilled water was added to the wells to release intracellular bacteria from the host cells. The colony-forming units (CFUs) were determined by plating dilutions on (buffered charcoal yeast extract (BCYE) agar plates. To each of the wells, 0.5 ml of fresh tissue culture medium was added, and the intracellular and extracellular bacteria in each well were combined at 24-h intervals. The total number of CFUs was determined by plating the dilutions onto BCYE agar plates.

The J774 cell monolayers were prepared on cover slips by the same procedures as described above. The cells were infected with *L. pneumophila* philadelphia-1 and other strains. After 48 h of infection, the infected J774 cells were stained by Gimenez staining and observed under a light microscope.

### Nucleotide sequence accession numbers

This Whole Genome Shotgun project has been deposited at GenBank under the Bioproject ID PRJNA281151, accession LAVP00000000-LBMS00000000 (Table S3).

## Additional Information

**How to cite this article**: Qin, T. *et al.* Population structure and minimum core genome typing of *Legionella pneumophila*. *Sci. Rep.* **6**, 21356; doi: 10.1038/srep21356 (2016).

## Supplementary Material

Supplementary Information
