# Supplementary material for: Population structure and minimum core genome typing of Legionella pneumophila
Source: Sci Rep. 2016 Feb 18;6:21356. doi: 10.1038/srep21356 (PMC4766850; doi:10.1038/srep21356)
Supplement: Supplementary Information [file srep21356-s1.pdf]

Population structure and minimum core genome typing of *Legionella pneumophila*

Tian Qin, Wen Zhang, Wenbin Liu, Haijian Zhou, Hongyu Ren, Zhujun Shao , Ruiting Lan, Jianguo Xu

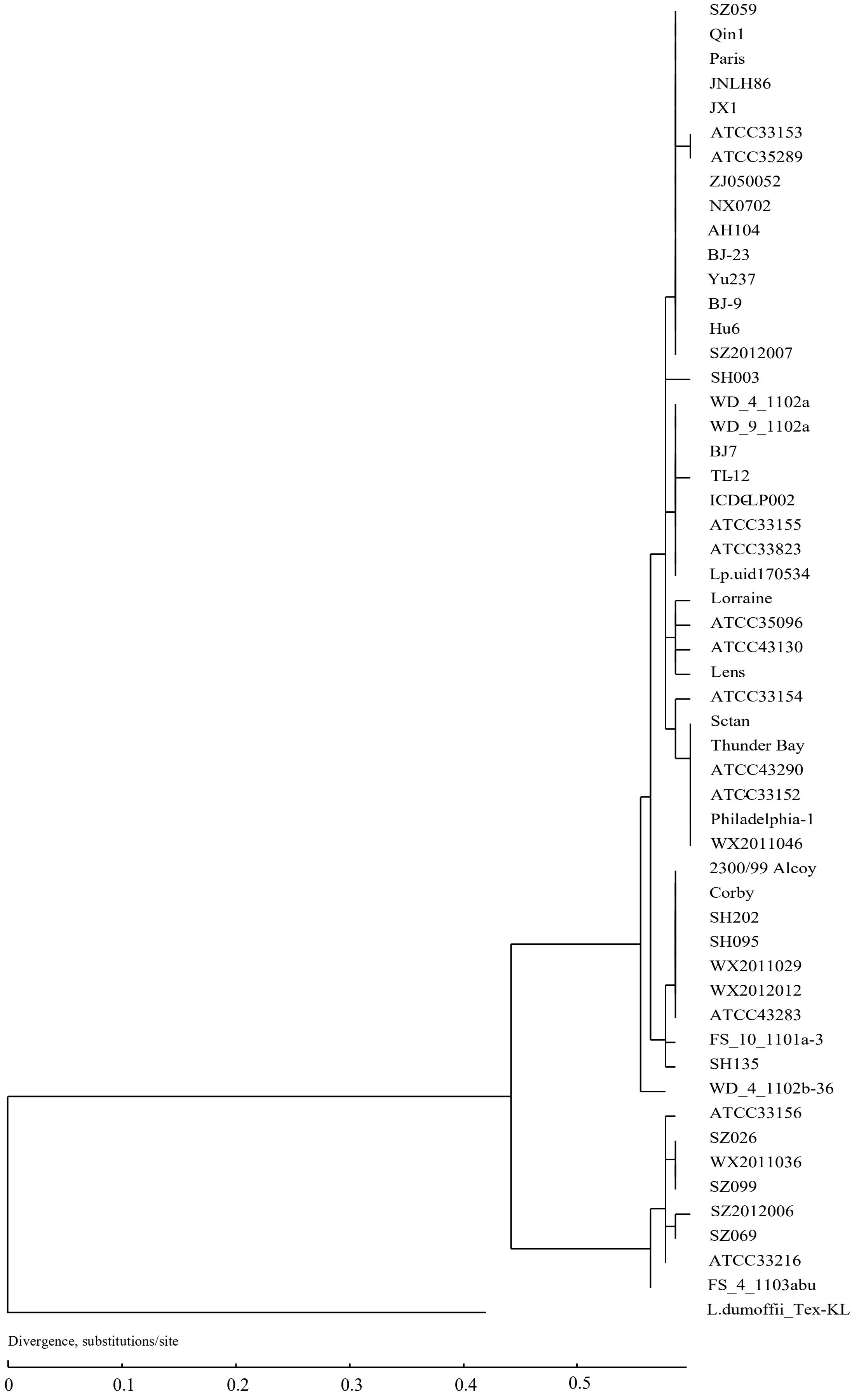

Figure S1. Maximum likelihood tree of 53 *L. pneumophila* strains based on single-copy orthologous genes using *Legionella dumoffii* strain Tex-KL as an outbreak

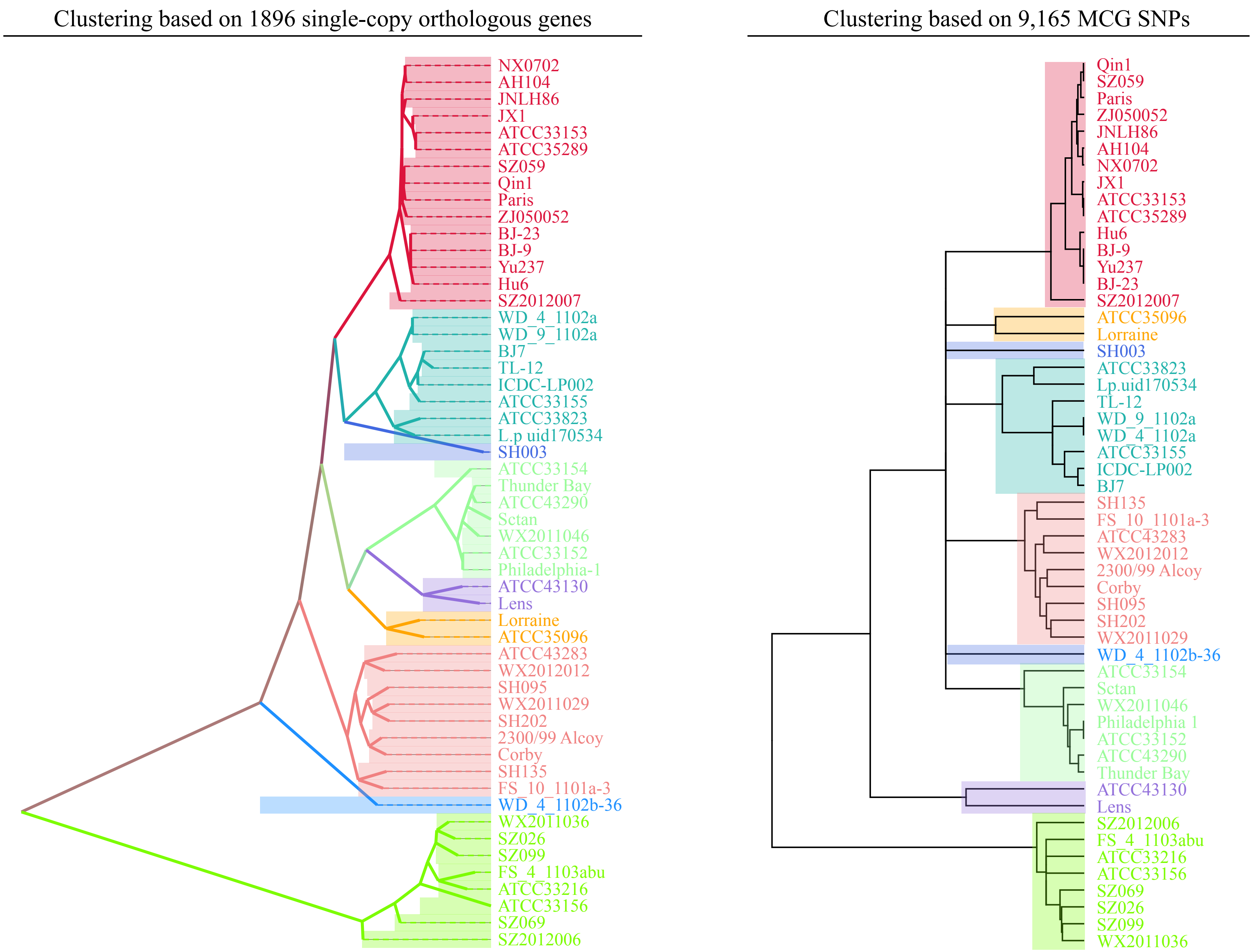

Figure S2. Phylogenetic analysis and grouping of 53 *L.pneumophila* strains based on 1896 single-copy orthologous genes and 9,165 MCG SNPs respectively. Nine groups were observed by both methods. The same group is shaded using same color.

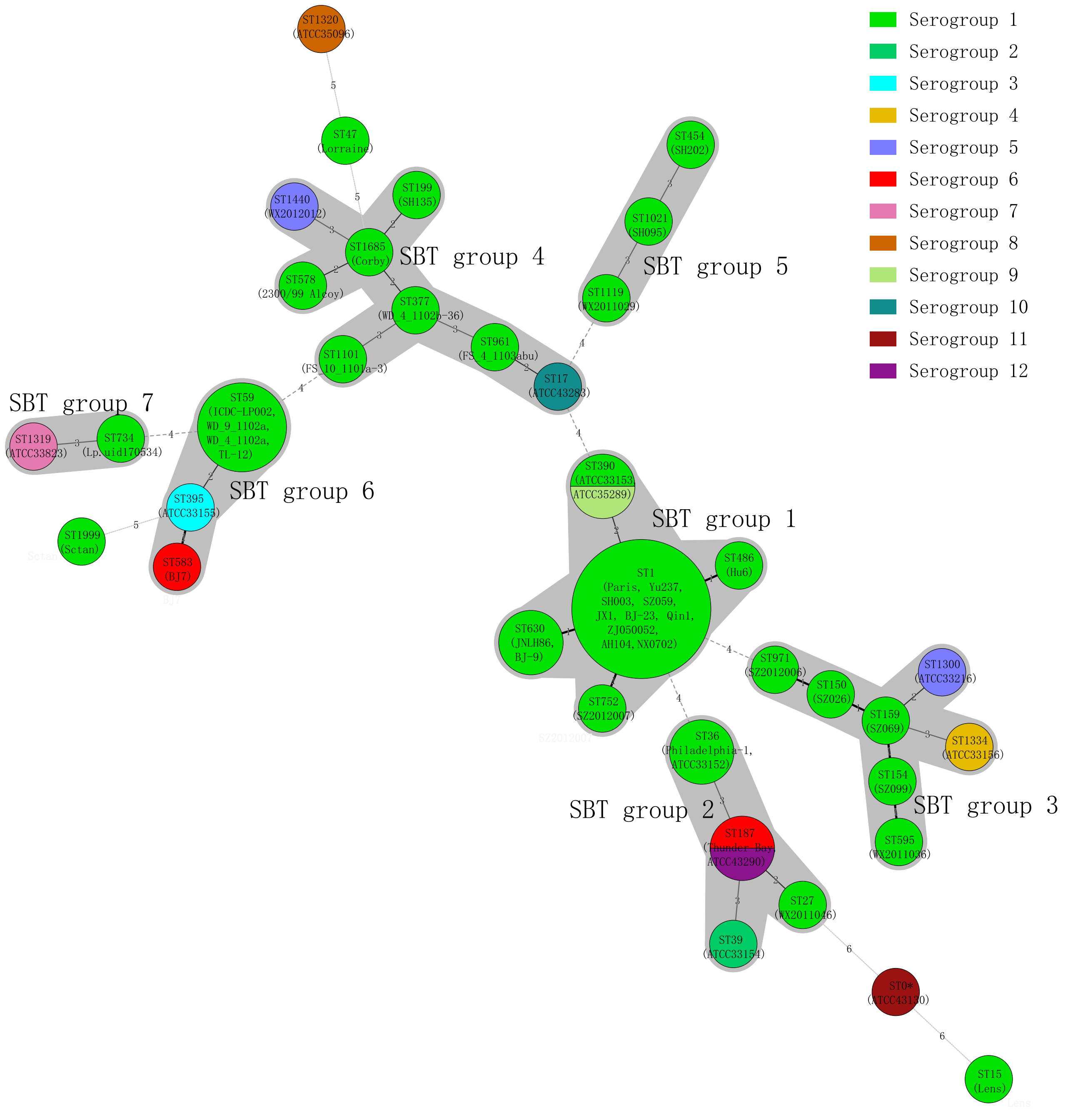

Figure S3. Minimum spanning tree of 53 *L.pneumophila* serogroup 1 strains. STs are shown as circles. The size of each circle indicates the number of isolates within this particular type; the STs and the strain ID in each ST are shown in the circles. The numbers of different loci between STs were showed in the lines linking two circles. The colors of the halo surrounding the STs represent types that belong to the same clonal group.

Table S1. Sequencing information of 53 *L.pneumophila* strains

Strain ID	Accession Number	GenomeSize (bp)	GC (%)	#Scaffold	#Gene	GeneSize (nt)	Coverage
Philadelphia-1	NC_002942	3397754	38.27	1	2943	3012525	0.886622
Lp.uid170534	NC_018140	3492535	38.35	1	3059	3044475	0.871709
Thunder Bay	CP003730.1	3455167	38.23	1	2998	3041772	0.880355
Paris	NC_006368	3635495	38.34	2	3224	3209355	0.882784
Lorraine	NC_018139	3467254	38.36	1	3069	3033201	0.874814
Lens	NC_006369	3405519	38.42	2	3004	3004116	0.882132
Corby	NC_009494	3576470	38.48	1	3204	3113565	0.870569
ATCC43290	NC_016811	3359001	38.17	1	2926	2993244	0.891111
2300/99 Alcoy	NC_014125	3516334	38.38	1	3190	3085662	0.877522
Yu237	LAVP00000000	3656267	38.26	46	3427	3221715	0.881149
SZ2012006	LAVQ00000000	3442741	38.18	32	3211	3041502	0.883454
SH003	LAVR00000000	3562062	38.14	60	3372	3141591	0.881959
SZ059	LBMR00000000	3610860	38.28	56	3368	3171039	0.878195
JNLH86	LAXO00000000	3711534	38.31	41	3490	3262551	0.87903
ATCC33153	LAXP00000000	3569569	38.22	45	3349	3144624	0.880953
JX1	LAXU00000000	3649843	38.23	40	3411	3219330	0.882046
BJ-23	LAXV00000000	3661813	38.25	45	3429	3223395	0.880273
SZ099	LAXW00000000	3426496	38.18	28	3205	3025542	0.882984
ICDC-LP002	LAXY00000000	3264206	38.14	41	3017	2867031	0.878324
Qin1	LAXZ00000000	3645343	38.23	43	3406	3216459	0.882347
SH135	LAYB00000000	3534089	38.2	80	3344	3093348	0.875289

ZJ050052	LAZZ00000000	3666043	38.23	55	3455	3223878	0.879389
FS_10_1101a-3	LBAA00000000	3386329	38.21	30	3158	2978211	0.879481
WD_9_1102a	LBAB00000000	3242781	38.13	39	3010	2857332	0.881136
SH202	LBMS00000000	3684811	38.26	90	3485	3218283	0.873392
SH095	LBAC00000000	3431853	38.18	79	3190	3013038	0.877962
ATCC33152	LBAD00000000	3308969	38.22	27	3083	2925582	0.884137
WD_4_1102a	LBAE00000000	3251458	38.15	42	3008	2858346	0.879097
SZ026	LBHM00000000	3473919	38.16	28	3246	3069768	0.883661
SZ069	LBAF00000000	3442598	38.18	38	3205	3039603	0.882939
TL-12	LBAG00000000	3427391	38.17	42	3191	3009513	0.878077
AH104	LBAJ00000000	3544661	38.25	37	3301	3127365	0.882275
Hu6	LBHL00000000	3553824	38.29	40	3311	3131109	0.881053
WX2011046	LBAK00000000	3437434	38.29	27	3200	3025992	0.880305
WX2011029	LBAL00000000	3591290	38.2	66	3401	3141249	0.874685
FS_4_1103abu	LBAM00000000	3543064	38.19	35	3334	3131571	0.88386
WD_4_1102b-36	LBAN00000000	3332434	38.29	44	3145	2931123	0.879574
SZ2012007	LBAP00000000	3597996	38.26	49	3345	3161850	0.878781
NX0702	LBAR00000000	3684924	38.21	52	3466	3249219	0.88176
WX2011036	LBAS00000000	3432941	38.2	30	3189	3032916	0.883475
BJ-9	LBAT00000000	3556240	38.28	44	3321	3130044	0.880155
Sctan	LAYA00000000	3364106	38.25	35	3123	2976411	0.884755
ATCC43283	LAXR00000000	3431441	38.3	18	3243	3034239	0.884246
ATCC43130	LAXS00000000	3286842	38.26	47	3139	2907555	0.884604
ATCC33154	LBAI00000000	3247985	38.2	44	3038	2879556	0.886567
ATCC33155	LBAO00000000	3407801	38.22	40	3168	3000489	0.880477
ATCC33156	LBAH00000000	3468720	38.18	49	3261	3067347	0.884288

ATCC33216	LBAV00000000	3456885	38.15	33	3223	3052245	0.882947
WX2012012	LBAU00000000	3493334	38.15	31	3280	3081330	0.88206
BJ7	LBAQ00000000	3475487	38.23	44	3236	3058170	0.879926
ATCC33823	LAXX00000000	3303037	38.2	46	3066	2898828	0.877625
ATCC35096	LAXT00000000	3338544	38.36	53	3112	2957415	0.88584
ATCC35289	LAXQ00000000	3567177	38.22	45	3338	3141546	0.880681

Table S2. Annotation and alleles of 25 marker genes identified to distinguish groups

Description	No. of alleles	Alleles								
		Group1	Group2	Group3	Group4	Group5	Group6	Group7	Group8	Group9
chromosomal replication initiation protein	4	1	2	3	1	1	1	1	1	4
hfq host factor-I protein for bacteriophage Q beta replication	2	1	1	1	1	1	1	1	1	2
hypothetical protein	9	1	2	3	4	5	6	7	8	9
large subunit ribosomal protein L7/L12	3	1	2	1	1	1	1	1	1	3
hypothetical protein	2	1	1	1	1	1	1	1	2	1
30S ribosomal protein S5	2	1	1	1	1	1	1	1	1	2
small subunit ribosomal protein S13	3	1	1	1	1	1	1	1	2	3
hypothetical protein conserved within Legionellae	2	1	1	1	1	1	1	1	1	2
glutathione synthase, ribosomal protein S6 modification protein	2	1	2	1	1	1	1	1	1	1
IcmL protein	4	1	2	1	1	1	1	1	3	4
membrane protein	3	1	1	1	1	1	1	1	2	3
flagellar basal body rod protein FlgG	3	1	2	1	1	1	3	1	1	1
ribosome-recycling factor	3	1	1	2	2	2	2	2	3	2
hypothetical protein	4	1	2	3	2	2	2	2	2	4
SecYEG protein translocase auxillary subunit	2	1	1	1	1	1	1	1	1	2
18K peptidoglycan-associated outer membrane lipoprotein	2	1	1	1	1	1	1	1	2	1
30S ribosomal protein S21	2	1	1	1	1	1	1	1	2	2

conserved protein of unknown function	2	1	2	1	1	1	1	1	1	1
peptide deformylase	3	1	2	1	1	1	1	1	1	3
membrane protein	6	1	2	1	3	1	1	4	5	6
UDP-3-O-[3-hydroxymyristoyl] N-acetylglucosamine deacetylase	4	1	2	1	1	3	1	1	4	4
peptidase M23	4	1	1	1	2	3	1	1	1	4
putative stringent starvation protein B	5	1	2	3	3	3	3	3	4	5
oligoribonuclease	4	1	2	1	1	1	1	1	3	4
hypothetical protein	2	1	1	1	2	1	2	1	1	1

Table S3. Groups obtained by Structure analysis

Group	Strain name	Strain number
1	Qin1, ATCC33153, Yu237, BJ-23, BJ-9, ZJ050052, JX1, JNLH86, Hu6, NX0702, ATCC35289, AH104, SZ2012007, Paris, SZ059	15
2	TL-12, WD_9_1102a, ATCC33823, WD_4_1102a, ATCC33155, ICDC-LP002, BJ7	7
3	SH003	1
4	ATCC43290, WX2011046, Thunder Bay, ATCC33154, Philadelphia-1, Sctan, ATCC33152	7
5	Lens	1
6	Lorraine	1
7	FS_10_1101a-3, SH202, SH095, 2300/99 Alcoy, ATCC43283, Corby, SH135, WX2012012, WX2011029	9
8	WD_4_1102b-36	1
9	SZ069, SZ2012006, ATCC33216, ATCC33156, SZ026, WX2011036, FS_4_1103abu, SZ099	8
Ungroup	ATCC43130, ATCC35096, Lp.uid170534	3

Table S4. Genetic Distance within and between minimal core genome groups

	MCGG1	MCGG2	MCGG3	MCGG4	MCGG5	MCGG6	MCGG7	MCGG8	MCGG9
MCGG1	0.012								
MCGG2	0.111	0.041							
MCGG3	0.176	0.174	n/c						
MCGG4	0.108	0.106	0.173	0.022					
MCGG5	0.177	0.179	0.248	0.125	0.095				
MCGG6	0.128	0.146	0.216	0.114	0.157	0.071			
MCGG7	0.113	0.109	0.195	0.107	0.180	0.147	0.040		
MCGG8	0.205	0.198	0.271	0.173	0.252	0.227	0.181	n/c	
MCGG9	0.263	0.247	0.317	0.204	0.265	0.271	0.254	0.297	0.031

The number of base substitutions per site from averaging over all sequence pairs within and between each group and between groups are shown. Analyses were conducted using the Maximum Composite Likelihood model [1]. The analysis involved 53 nucleotide sequences. All positions containing gaps and missing data were eliminated. There were a total of 9165 positions in the final dataset. Evolutionary analyses were conducted in MEGA5 [2]. The presence of n/c in the results denotes cases in which it was not possible to estimate evolutionary distances.

1. Tamura K., Nei M., and Kumar S. (**2004**). Prospects for inferring very large phylogenies by using the neighbor-joining method. *Proceedings of the National Academy of Sciences* (*USA*) **101**:11030-11035.
2. Tamura K., Peterson D., Peterson N., Stecher G., Nei M., and Kumar S. (**2011**). MEGA5: Molecular Evolutionary Genetics Analysis using Maximum Likelihood, Evolutionary Distance, and Maximum Parsimony Methods. *Molecular Biology and Evolution* 28: 2731-2739.

Table S5. Lethal mutations (premature stop, damaged start codon, damaged stop codon) associating genes in the genomes of eight group 9 strains by comparing to the genome of Philadelphia 1 *

Ref gene	Mutate-type	Mutate-type detail	Product	BLAST results	ATCC 33156	ATCC33216	FS_4_1103abu	SZ026	SZ069	SZ099	SZ2012006	WX2011036
YP_094125.1	SNP	Premature_stop	hypothetical protein	Aspartate aminotransferase A (Transaminase A) (AspAT)	P	P	P	P	P	P	P	P
YP_094218.1	SNP	Damaged_Stop_Codon	hypothetical protein	alpha/beta fold family hydrolase	P	P	P	P	P	P	P	P
YP_094247.1	SNP	Premature_stop	hypothetical protein	catalase-peroxidase (katA) gene	P	P	P	P	P	P	P	P
YP_094412.1	SNP	Premature_stop	hypothetical protein	hypothetical protein	P	P	P	P	P	P	P	P
YP_094509.1	SNP	Premature_stop	hypothetical protein	3-dehydroquinate dehydratase II	P	P	P	P	P	P	P	P
YP_094553.1	SNP	Premature_stop	hypothetical protein	UDP-3-O-(3-hydroxymyristoyl)-glucosamine N-acyltransferase（lpxD）,	P	P	P	P	P	P	P	P
YP_094918.1	SNP	Premature_stop	hypothetical protein	Glycosyl hydrolase, family 3 beta-N-acetylhexosaminidase	P	P	P	P	P	P	P	P
YP_095041.1	SNP	Damaged_Start_Codon Damaged_Stop_Codon	cobalt/zinc/cadmium efflux RND transporter permease HelA	Cobalt-zinc-cadmium resistance protein CzcA;	P	P	N	P	P	P	P	P
YP_095115.1	SNP	Premature_stop	hypothetical protein	hypothetical protein	N	N	N	N	P	N	P	N
YP_095244.1	SNP, Indel	Premature_stop, Damaged_Start_Codon	hypothetical protein	Predicted ATPase	P	N	N	N	N	N	N	N
YP_095287.1	SNP	Damaged_Stop_Codon	hypothetical protein	LvrB2	P	P	P	N	P	P	P	P
YP_095305.1	SNP	Damaged_Stop_Codon	TrkA family protein (Transport and binding)	transporter, TrkA family	P	P	P	P	P	P	P	P
YP_095628.1	SNP	Premature_stop	hypothetical protein	hypothetical protein	P	P	N	P	P	P	P	P

YP_096130.1	SNP	Premature_stop	transposase TnpA (Viral functions / Phage / Transposases)	transposase TnpA	N	N	N	P	N	N	N	
YP_096155.1	SNP, Indel	Premature_stop	hypothetical protein	soluble lytic murein transglycosylase	P	P	P	P	N	P	P	P
YP_096207.1	Indel	Premature_stop	hypothetical protein	hypothetical protein	N	P	N	P	P	P	P	P
YP_096373.1	SNP, Indel	Premature_stop	hypothetical protein (Transport and binding, Protein fate /hydrolases / secretion, Viral functions /Phage /Transposases)	IncP-type oriT binding protein TraK	P	N	P	P	N	P	N	P
YP_096402.1	SNP	Damaged_Start_Codon	hypothetical protein	hypothetical protein	N	P	P	P	P	N	P	
YP_096798.1	SNP	Premature_stop	hypothetical protein	Lgn1-1	P	P	N	P	P	P	P	P

* P, positive; N, negative

Table S6. Serogroup, epidemiological and SBT data of 53 *L.pneumophila* strains

Strain ID	Serogroups	Isolated country	Isolated year	Source	SBT type	SBT cluster
Philadelphia-1	LP1	USA	Unknown	Human lung	ST36	SG2
Lp.uid170534	LP1	USA	Unknown	Unknown	ST734	SG7
Thunder Bay	LP6	Canada	Unknown	Clinical sample	ST187	SG2
Paris	LP1	France	Unknown	Unknown	ST1	SG1
Lorraine	LP1	Unknown	Unknown	Unknown	ST47	Single
Lens	LP1	France	Unknown	Unknown	ST15	Single
Corby	LP1	Unknown	Unknown	Unknown	ST1685	SG4
ATCC43290	LP12	Unknown	Unknown	Clinical sample	ST187	SG2
2300/99 Alcoy	LP1	Unknown	Unknown	Unknown	ST578	SG4
Yu237	LP1	China	2005	Cooling tower water	ST1	SG1
SZ2012006	LP1	China	2012	Hot spring water	ST971	SG3
SH003	LP1	China	2008	Cooling tower water	ST1	SG1
SZ059	LP1	China	2005	Cooling tower water	ST1	SG1
JNLH86	LP1	China	2009	Tap water	ST630	SG1
ATCC33153	LP1	USA	Unknown	Clinical sample	ST390	SG1
JX1	LP1	China	2008	Cooling tower water	ST1	SG1
BJ-23	LP1	China	2006	Cooling tower water	ST1	SG1
SZ099	LP1	China	2005	Cooling tower water	ST154	SG3
ICDC-LP002	LP1	China	2012	Clinical sample	ST59	SG6
Qin1	LP1	China	2008	Cooling tower water	ST1	SG1
SH135	LP1	China	2008	Cooling tower water	ST199	SG4
ZJ050052	LP1	China	2006	Cooling tower water	ST1	SG1
FS_10_1101a-3	LP1	China	2011	Hot spring water	ST1101	SG4

WD_9_1102a	LP1	China	2011	Hot spring water	ST59	SG6
SH202	LP1	China	2008	Cooling tower water	ST454	SG5
SH095	LP1	China	2008	Cooling tower water	ST1021	SG5
ATCC33152	LP1	USA	Unknown	Clinical sample	ST36	SG2
WD_4_1102a	LP1	China	2011	Hot spring water	ST59	SG6
SZ026	LP1	China	2005	Cooling tower water	ST150	SG3
SZ069	LP1	China	2005	Cooling tower water	ST159	SG3
TL-12	LP1	China	2005	Hot spring water	ST59	SG6
AH104	LP1	China	2006	Cooling tower water	ST1	SG1
Hu6	LP1	China	2008	Cooling tower water	ST486	SG1
WX2011046	LP1	China	2010	Cooling tower water	ST27	SG2
WX2011029	LP1	China	2010	Cooling tower water	ST1119	SG5
FS_4_1103abu	LP1	China	2011	Hot spring water	ST961	SG4
WD_4_1102b-36	LP1	China	2011	Hot spring water	ST377	SG4
SZ2012007	LP1	China	2012	Hot spring water	ST752	SG1
NX0702	LP1	China	2007	Cooling tower water	ST1	SG1
WX2011036	LP1	China	2010	Cooling tower water	ST595	SG3
BJ-9	LP1	China	2005	Cooling tower water	ST630	SG1
Sctan	LP1	China	2005	Clinical sample	ST1999	Single
ATCC43283	LP10	the Netherlands	Unknown	Clinical sample	ST17	SG4
ATCC43130	LP11	Unknown	Unknown	Clinical sample	ST0	Single
ATCC33154	LP2	Unknown	Unknown	Clinical sample	ST39	SG2
ATCC33155	LP3	Unknown	Unknown	Creek water	ST395	SG6
ATCC33156	LP4	USA	1988	Clinical sample	ST1334	SG3
ATCC33216	LP5	Unknown	Unknown	Cooling tower	ST1300	SG3
WX2012012	LP5	China	2012	Clinical sample	ST1440	SG4

BJ7	LP6	China	2004	Domestic hot water	ST583	SG6
ATCC33823	LP7	Unknown	Unknown	Showerhead	ST1319	SG7
ATCC35096	LP8	USA	Unknown	Clinical sample	ST1320	Single
ATCC35289	LP9	the Netherlands	Unknown	Tap water	ST390	SG1

Table S7. Ln probability of 53 strains using Structure from K=1 to K20 (BURNIN=50000 and NUMREPS=150000)

	Ln Prob					
k	1	2	3	4	5	average
1	-146481.7	-146754.8	-146481.6	-146481.7	-146481.7	-146536.3
2	-120006.4	-104928.0	-115648.1	-115647.1	-104926.7	-112231.26
3	-81158.4	-81151.4	-81158.0	-81151.5	-81157.7	-81155.4
4	-67520.5	-73393.5	-67510.3	-67522.4	-67512.2	-68691.78
5	-62261.9	-57160.5	-62383.8	-62288.3	-62578.2	-61334.54
6	-52178.2	-58959.1	-68465.8	-52190.0	-58940.6	-58146.74
7	-48715.7	-48739.8	-55381.2	-57851.0	-48703.8	-51878.3
8	-56601.9	-58967.6	-52085.5	-55169.6	-57571.2	-56079.16
9	-52528.7	-51497.9	-56484.7	-42709.5	-44260.0	-49496.16
10	-50137.6	-44256.3	-48725.5	-51345.2	-54433.1	-49779.54
11	-54744.9	-41188.9	-40309.5	-41644.6	-43875.2	-44352.62
12	-40558.8	-40537.8	-44996.4	-54296.7	-42017.2	-44481.38
13	-40675.5	-46016.1	-41104.5	-40051.8	-42105.3	-41990.64
14	-52242.8	-40651.7	-40609.8	-40205.7	-40946.5	-42931.3
15	-40880.8	-46980.4	-40005.5	-43010.2	-37708.1	-41717
16	-43829.8	-50781.3	-50468.4	-40638.3	-35611.8	-44265.92
17	-42144.0	-40881.3	-39671.9	-40286.1	-39544.0	-40505.46
18	-47987.6	-50944.8	-36331.9	-50739.8	-49042.4	-47009.3
19	-36320.1	-36580.1	-42238.9	-40037.1	-37272.2	-38489.68
20	-38589.9	-36831.0	-39929.6	-39976.0	-36914.7	-38448.24
